# Quantitative Analysis of Cenobamate and Concomitant Anti-Seizure Medications in Human Plasma via Ultra-High Performance Liquid Chromatography–Tandem Mass Spectrometry

**DOI:** 10.3390/molecules29040884

**Published:** 2024-02-17

**Authors:** Linda Molteni, Bruno Charlier, Albino Coglianese, Viviana Izzo, Giovanni Assenza, Pierantonio Menna, Ugo de Grazia, Annachiara D’Urso

**Affiliations:** 1 SSD Laboratory Medicine, Fondazione IRCCS “Istituto Neurologico Carlo Besta”, 20133 Milan, Italy; ugo.degrazia@virgilio.it; 2 Department of Medicine, Surgery and Dentistry “Scuola Medica Salernitana”, University of Salerno, Baronissi, 84081 Salerno, Italy; bcharlier@unisa.it (B.C.); albino.cog@gmail.com (A.C.); vizzo@unisa.it (V.I.); 3 University Hospital “San Giovanni di Dio e Ruggi d’Aragona”, 84131 Salerno, Italy; 4 Graduate School in Clinical Pathology and Clinical Biochemistry, University of Salerno, Baronissi, 84081 Salerno, Italy; 5 Fondazione Policlinico Universitario Campus Bio-Medico, 00128 Rome, Italy; g.assenza@policlinicocampus.it (G.A.); p.menna@policlinicocampus.it (P.M.); 6 Department of Science and Technology for Sustainable Development and One Health, University Campus Biomedico di Roma, 00128 Rome, Italy

**Keywords:** cenobamate, anti-seizure medications, therapeutic drug monitoring, pharmacokinetics, UHPLC–MS/MS

## Abstract

Cenobamate (CNB) is a new anti-seizure medication (ASM) recently introduced in clinical practice after approval by the FDA and EMA for the add-on treatment of focal onset seizures in adult patients. Although its mechanism of action has not been fully understood, CNB showed promising clinical efficacy in patients treated with concomitant ASMs. The accessibility of CNB could pave a way for the treatment of refractory or drug-resistant epilepsies, which still affect at least one-third of the patients under pharmacological treatment. In this context, therapeutic drug monitoring (TDM) offers a massive opportunity for better management of epileptic patients, especially those undergoing combined therapy. Here, we describe the first fully validated ultra-high performance liquid chromatography-tandem mass spectrometry (UHPLC–MS/MS) method for the quantification of CNB and concomitant ASMs in human plasma, with samples extracted either manually or by means of a liquid handler. Our method was validated according to the most recent ICH International Guideline M10 for Bioanalytical Method Validation and Study Sample Analysis. The method proved to be selective for CNB and displayed a linear range from 0.8 to 80 mg/L; no matrix effect was found (98.2 ± 4.1%), while intra-day and inter-day accuracy and precision were within the acceptance range. Also, CNB short- and long-term stability in plasma under different conditions was assessed. Leftover human plasma samples were employed as study samples for method validation. Our method proved to be highly sensitive and selective to quantify CNB and concomitant ASMs in human plasma; therefore, this method can be employed for a routinely TDM-based approach to support physicians in the management of an epileptic patient.

## 1. Introduction

Epilepsy is one of the most common and heterogeneous neurological disorders, affecting about 70 million people worldwide. It is estimated that in industrialized countries almost 3–4% of people will develop epilepsy during their lifetime; the risk is higher in developing countries, exacerbating the deleterious effects on physical, psychological, and social well-being [1,2]. 

The treatment of epilepsies is merely symptomatic, with no currently available therapies able to correct the underlying disorder, instead only aimed at suppressing seizure manifestation [3]. For this reason, people with epilepsies will need a lifelong treatment, characterized by frequent changes in medicaments and polytherapy [1,3,4].

The administration of anti-seizure medications (ASMs) is considered the mainstay treatment against epilepsies [1,4]; even though novel ASMs have been introduced in recent years, along with first-, second-, and third-generation drugs already available for pharmacological treatment, there is no evidence of any improvement in treatment outcomes in patients with newly diagnosed epilepsies within the past two decades [3,5].

In a previous study, it was found that barely two-thirds of the patients with newly diagnosed epilepsies have reached seizure freedom, intended as 100% seizure reduction after ASMs treatment, while one-third of patients continued to have partial seizure control. In particular, those who did not respond to the first or second ASM regimen were more prone to develop refractory or drug-resistant epilepsies [6,7]. It has also been shown that the probability of becoming seizure-free decreases with subsequent ineffective ASM treatments [3,5]. In this scenario, it is clear that refractory epilepsies still represent a great challenge, with an unmet need for new treatment strategies aimed at ensuring long-term seizure control [8].

**Figure 1:**
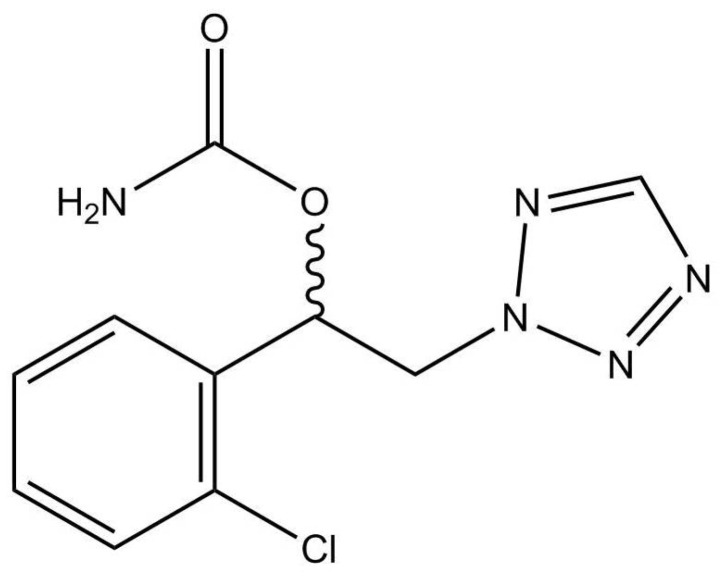
Chemical structure of CNB.

Cenobamate (CNB) ([(*R*)-1-(2-chlorophenyl)-2-(2*H*-tetrazol-2-yl)ethyl]) is a tetrazole alkyl carbamate derivative (Figure 1), recently introduced in clinical practice as an ASM. In November 2019, the Food and Drug Administration (FDA) approved XCOPRI^®^, marketed by SK Life Science Inc. (Paramus, NJ, USA) for the adjunctive treatment of focal onset seizures with or without secondary generalization in adult patients. Afterwards, in March 2021, the European Medicines Agency (EMA) approved Ontozry^®^, marketed by Arvelle Therapeutics Netherlands B.V. (Amsterdam, The Netherlands) [9,10].

CNB’s short-term efficacy was demonstrated in two randomized, double-blind, multicenter, placebo-controlled clinical trials (YKP3089C013; NCT01397968 and YKP3089C017; NCT01866111) in which CNB was administrated once daily as an adjunctive treatment in adults with drug-resistant focal seizures. It was found that CNB reduced seizure frequency by at least 50% in 50.1% of the patients randomized to receive CNB adjunctively to their existing ASMs, compared to placebo and in a dose-dependent manner. In addition, seizure freedom rates were higher and dose-dependent in the CNB group compared to placebo, with 27.5% of patients achieving seizure freedom in the first trial [11,12,13,14]. Subsequent open-label extended clinical trials demonstrated that seizure freedom was maintained for up to 30 months [15]. CNB was well tolerated, with few central adverse events mainly due to the rate of the titration process (including dizziness, insomnia, diplopia, disturbances in gait and coordination) [16,17].

CNB showed a more favorable relationship between efficacy and tolerability when compared with other ASMs, suggesting that it may have a different and unique mechanism of action responsible for its clinical efficacy [9,18]. The precise mechanism of action of CNB has not been established yet, but in vitro data suggest that CNB might regulate both excitatory and inhibitory transmission. Nakamura et al. have shown that CNB targets voltage-gated sodium channels and inhibits the persistent component of the sodium ion current (I_NaP_) in hippocampal CA3 neurons, thus, hindering repetitive firing [19]. Moreover, Sharma et al. have demonstrated that CNB acts as a positive allosteric modulator of human γ-aminobutyric acid (GABA) receptors by binding to non-benzodiazepine sites, hence, regulating both phasic and tonic currents [20].

In this context, CNB’s suggested mechanism of action represents a novelty in the sphere of ASMs; only phenytoin (PHT) shares a similar action on I_NaP_. However, despite this analogy, PHT shows a narrower anti-seizure profile when compared to CNB. Moreover, the more conventional sodium-blocker ASMs act on the transient component of the sodium ion currents (I_NaT_), which is primarily responsible for the rapid depolarization of the action potential, rather than on I_NaP_, which is suggested to be involved in the amplification of subthreshold synaptic potentials and the facilitation of repetitive firing in neurons [21]. This implies that the inhibition of I_NaP_ could play a crucial but not sufficient role in order to explain CNB’s clinical efficacy. The combination of both excitatory and inhibitory regulation is thought to contribute to the broad anti-seizure spectrum of CNB and could explain its promising efficacy [9,18].

In the abovementioned clinical trials, CNB was administered as an adjunctive treatment with one to three other concomitant ASMs. Thus, it is of crucial importance to evaluate both pharmacokinetics (PK) and pharmacodynamics (PD) interactions between CNB and co-administered ASMs [16]. After a single oral administration, CNB is rapidly adsorbed (t_max_ = 1–4 h), showing high bioavailability (88%), and is moderately bound to plasma proteins (60%). Its plasma exposure increases with drug concentration, with a more than proportional increase at high doses. CNB showed a long half-life (t_1/2_ = 50–60 h) within the therapeutic dose range (100–400 mg/die). The prolonged t_1/2_ and the more than proportional plasma exposure of CNB at high doses suggest a non-linear process of distribution and elimination. CNB is primarily metabolized in the liver by both phase II and phase I reactions, the latter carried out by several cytochrome P450 (CYP) isoforms, including CYP2E1, CYP2A6, CYP2B6, CYP2C19, and CYP3A4/5. As a CYP substrate, CNB affects CYP activity by inhibiting or inducing different CYP isoenzymes, including CYP2C19 or CYP2C8. Interestingly, it can also inhibit or induce CYP2B6 and CYP3A4 isoforms. No effect on transporter proteins has been shown [10,22].

Several studies were carried out to assess drug–drug interactions (DDIs) between CNB and other concomitant ASMs. It was shown, for example, that PHT and phenobarbital (PB) reduced the area under the curve (AUC) of CNB; interestingly, by inhibiting CYP2C19, CNB was also able to increase plasma concentrations of PB, PHT, brivaracetam (BRV), and cannabidiol. Moreover, by inducing CYP3A4, CNB was able to reduce the plasma concentrations of lamotrigine (LTG) and carbamazepine (CBZ). A decrease in plasma concentrations of clonazepam, ethosuximide (ETS), felbamate, midazolam, perampanel (PMP), and zonisamide (ZNS) is also expected. CNB interactions were assessed on other types of drugs, including omeprazole, oral contraceptives, and bupropion, following the same mechanisms [10,16]. Despite this preliminary evidence, more extensive clinical studies will be needed to better clarify both DDIs induced by CNB and, most importantly, its mechanism of action, which has not been fully characterized yet. 

As previously mentioned, CNB represents an add-on therapy to concomitant ASMs; therefore, its introduction in routine therapeutic drug monitoring (TDM), as already performed for the other ASMs, could be of crucial advantage. The measurement of CNB plasma concentrations might allow for a better definition of therapeutic ranges and give pivotal information on its PK under different conditions and when co-administered with other ASMs. TDM of CNB could also help with concerns related to patient management in terms of efficacy and safety by identifying a personalized therapeutic plan.

Here, we describe an update of our previously validated method [23] for the simultaneous quantification of CNB and concomitant ASM plasma concentrations, including pregabalin (PGB), gabapentin (GBP), levetiracetam (LEV), ethosuximide (ETS), lamotrigine (LTG), primidone (PRM), lacosamide (LCS), zonisamide (ZNS), rufinamide (RUF), 10-OH-monohydroxycarbazepine (10-OH-MHD), brivaracetam (BRV), carbamazepine-epoxide (CBZ-E), topiramate (TPM), tiagabine (TGB), perampanel (PMP), and stiripentol (STP). CBZ, PB, PHT, and valproic acid (VPA) were excluded from this study, as in our laboratories these drugs are routinely monitored by immunochemistry assays.

Our UHPLC–MS/MS analytical method was validated according to the most recent ICH Guideline M10 on Bioanalytical Method Validation and Study Sample Analysis [24]. To the best of our knowledge, this represents the first fully validated UHPLC–MS/MS method for the simultaneous quantification of CNB and other ASMs in human plasma samples.

## 2. Results and Discussion

### 2.1. Comparison of Existing Methods

Thus far, only a few analytical methods have been developed for the quantification of CNB in plasma samples (Table 1). Oh et al. first developed a method for the quantification of CNB in rat plasma, paving the way for the determination of CNB using LC-MS/MS [25]. This method employed a simple protein precipitation extraction with carisbamate as internal standard and showed a short run time; however, the calibration range was quite limited (0.01–5 mg/L). 

During phase 1 and phase 2 clinical studies, human plasma samples were analyzed to validate an LC-MS/MS method for the quantification of CNB; however, methodological and analytical procedures were not reported in detail [22]. The calibration range was limited (0.02–10 mg/L) and a simple protein precipitation extraction was employed; phenacetin was used as internal standard. 

Other LC-MS/MS methods in human plasma were developed and applied for pharmacokinetic studies of CNB with both a single-ascending dose (SAD) and multiple-ascending dose (MAD) [26]. The first method, which was employed for both SAD and MAD studies, showed a calibration range from 0.02 to 25 mg/L; CNB was extracted with a simple protein precipitation extraction with acetonitrile, and phenacetin was used as internal standard. The second method was employed for MAD studies only and showed a more limited calibration range (0.02–10 mg/L); the samples were precipitated with acetonitrile and the obtained supernatant was evaporated and reconstituted with acetonitrile and water. Phenacetin was once again used as internal standard. The third and last method described by Vernillet et al. [26], employed for MAD studies, showed a more extended calibration range (0.05–40 mg/L) and a more complicated extraction procedure was used; lipid extraction with methyl tertiary-butyl ether was performed and the supernatant was then evaporated and reconstituted with methanol and water. This time, d4-cenobamate was introduced as internal standard, thus allowing a more robust quantification.

Most recently, our group has developed and validated a UHPLC–MS/MS method for the monitoring of CNB plasma concentrations in two patients who underwent CNB dose titration and concomitant ASM regimens [23]. This method represented a fast, simple, and robust tool for the quantification of CNB in a calibration range from 0.05 to 20 mg/L. A simple protein precipitation extraction with acetonitrile and lamotrigine-C13-d3 as internal standard was performed. The chromatographic separation was fast, with a 3.5 min run time.

As a result of the introduction of CNB into a clinical practice in which several other ASMs are co-administered, the need to develop a single method for the simultaneous quantification of all other available ASMs has undoubtedly emerged. In this framework, our method provides a simple, robust, and rather fast quantification of CNB and 16 other ASMs within a single run. The plasma samples are extracted with a simple protein precipitation and, as in our previous method, lamotrigine-C13-d3 is used as internal standard. Employed by Vernillet et al. [26], d4-cenobamate represents the gold standard for the normalization of CNB signal, however, it is difficult to obtain; conversely, lamotrigine-C13-d3 is already used for the internal normalization of lamotrigine and was also found to provide good normalization capabilities for CNB. The chromatographic separation is simple and has a relatively short run time of 7 min. Moreover, the calibration range was extended from 0.80 to 80 mg/L, reflecting the plasma concentrations reported in the previous articles and those described by de Grazia et al. [27]. Finally, the simultaneous quantification of CNB and other ASMs within a single method allows us to save both reagent costs and time, also providing a tool for a more efficient clinical management of the patient.

### 2.2. Method Validation

The analytical method was fully validated for CNB, given that the quantification methods for the other compounds have been previously validated by the kit manufacturer and the CE/IVD kit for the quantification of other ASMs is already in use in our laboratory for routine TDM. Data on partial kit validation, as recommended by ICH Guideline M10 [24], for the quantification of concomitant ASMs are reported in Supplementary Material and are also discussed in a previous study [28].

**Figure 2:**
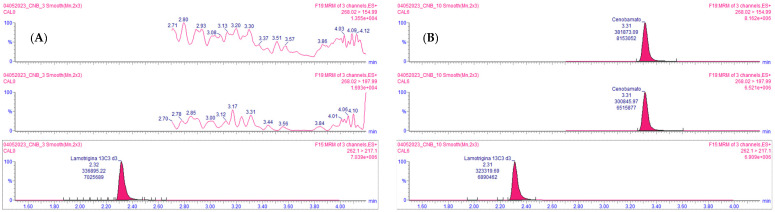
Representative CNB chromatograms in blank plasma samples extracted with internal standard (IS) (**A**), and in plasma samples spiked with CNB and extracted with IS (**B**).

The method proved to be selective and sensitive, since no chromatographic peaks from interfering components, including other ASMs, were observed, with a response higher than 20% of the lower limit of quantification (LLOQ) signal. Figure 2 shows representative chromatograms of blank plasma and samples spiked with CNB.

A seven-point calibration curve was built, as well as three concentration levels of quality control (QC) samples. The calibration range was determined based on a recent publication by de Grazia et al. [27], in which expected CNB plasma concentrations were reported within 5–60 mg/L. In our previous study, the calibration curve was limited to 20 mg/L; since the reported CNB plasma concentrations were higher, we decided to extend the calibration range above these values.

**Figure 3:**
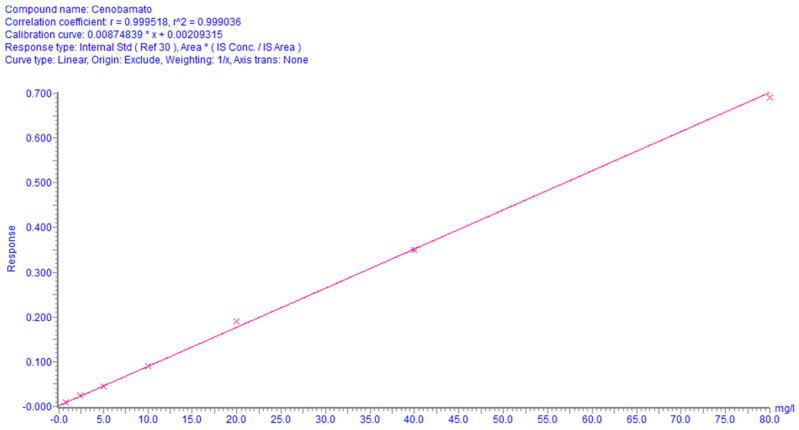
CNB calibration curve in plasma. The curve equation is y = 0.00874839x + 0.00209315. R^2^ is 0.999.

A 1/x weighted linear regression model was applied and the method showed good linearity over the calibration range, as shown in Figure 3. LLOQ was set at 0.8 mg/L for CNB and was included in the calibration curves, as well as for those of the other measured compounds (shown in Supplementary Material—Table S1).

No carry-over was observed: six blank injections of samples at high concentrations showed a mean signal of 8.3 ± 1.8% of LLOQ response for CNB.

Dilution integrity was assessed by measuring precision and accuracy of diluted samples (*n* = 5): mean bias% and CV% were, respectively, 9.5% and 4.0% for the 1:2 dilution factor and 10.5% and 3.7% for the 1:16 dilution factor.

No matrix effect (ME) could be found for CNB when measured at low QC (LQC) and high QC (HQC), while recovery (RE) was acceptable, as shown in Table 2.

Precision and accuracy were assessed at LLOQ, LQC, medium QC (MQC), and HQC, following a 5 × 5 scheme. Bias% and CV% values for inter-day and intra-day measurements are shown in Table 3. All values met the acceptance criteria.

Accuracy and precision were also assessed for the other measured compounds in order to evaluate the robustness of the bioanalytical method. Data are shown in Supplementary Material (Tables S2 and S3).

Reinjection reproducibility was evaluated by measuring accuracy and precision of a reinjected run. All values were within the acceptance criteria.

Stability was assessed under different storing conditions (Table 4). CNB in plasma proved to be stable up to 1 week at 4 °C, up to 1 month at −40 °C, and up to 24 h when stored in the autosampler compartment (10 °C). Freeze-thaw samples at −20 °C showed an overall good stability, as well as samples stored at −20 °C for 1 month. CNB concentration in plasma samples stored at room temperature (RT) for 72 h was shown to be highly unstable.

Incurred sample reanalysis (ISR) of study samples was performed on 15 out of the 29 samples included in the method validation; 80% of the repeated values showed a percentage difference below ±20% when compared to the initial values.

#### Automated Sample Extraction Validation

Partial method validation, according to the most recent ICH Guideline M10 for Bioanalytical Method Validation [24], was also carried out for CNB when an automated sample extraction procedure was employed.

No carry-over was observed: six blank samples dispensed after the handling of samples at high concentrations showed a mean signal of 3.5 ± 1.8% of CNB LLOQ response (3.4 ± 2.2% for the first handling tip and 3.5 ± 1.5% for the second handling tip).

No ME was found for CNB, while RE was acceptable. Internal standard normalized ME (ISn-ME) was 97.6 ± 5.9% for LQC and 101.5 ± 5.2% for HQC, while internal standard normalized RE (ISn-RE) was 94.0 ± 6.7% for LQC and 105.6 ± 3.8% for HQC.

### 2.3. Analysis of Patient Samples

The analysis of anonymized real samples is a necessary step in the validation process of a bioanalytical method, as reported in the ICH Guideline M10 [24]. Leftover plasma samples collected for routine TDM of ASMs from patients in co-therapy with CNB treated at Fondazione IRCCS Istituto Neurologico Carlo Besta were analyzed as study samples in order to assess method robustness. In total, 29 samples from 14 patients in co-therapy with CNB were collected and processed. Patients’ features are described in Supplementary Material (Table S4). 

Our patients are 60% male while 40% are female and their age ranges from 20 to 75 years old. The daily administered CNB dose ranges from 12.5 to 400 mg, as a reflection of the titration process; the measured CNB concentrations span from 0.9 to 54.2 mg/L, in agreement with the concentration ranges previously described [27]. No concentration values were found above the upper quantification limit of 80 mg/L, while a few samples were found below the LLOQ, even though patients were reported to consume CNB.

**Figure 4:**
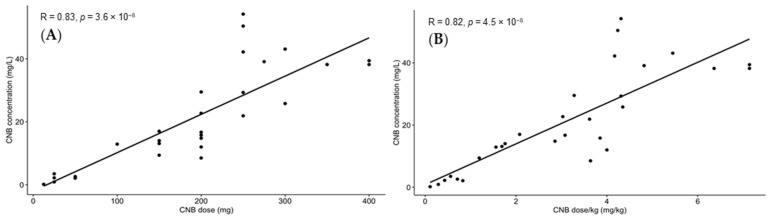
Significant (*p* < 0.0001) linear correlation (Pearson test) between daily dose and the plasma concentration of CNB (**A**), and between daily dose pro kg and the plasma concentration of CNB (**B**). Statistical analysis was performed with R software (version 4.3.2).

Our data suggest a linear PK of CNB, with a positive correlation between CNB daily dose and CNB plasma concentrations. A linear correlation was also reported when CNB daily dose pro kg was employed (Figure 4). Moreover, a high inter-subject variability is observed, presumably due to intrinsically different patient features and to the potential interactions with other concomitant therapies. Amongst our patients, the co-administered ASMs were VPA, CBZ, PB, TMP, BRIVA, LCS, PMP, clobazam, LEV, PGB, LMT, ZNS, and PHT; unfortunately, due to the small population, no considerations about DDIs reported among our patients can be drafted. In this context, as a result of the great inter-individual variability and of the strong influence of DDIs on CNB PK [10], the quantification of CNB plasma concentrations represents a necessary step for the identification of a personalized drug regimen.

### 2.4. Method Comparison

**Figure 5:**
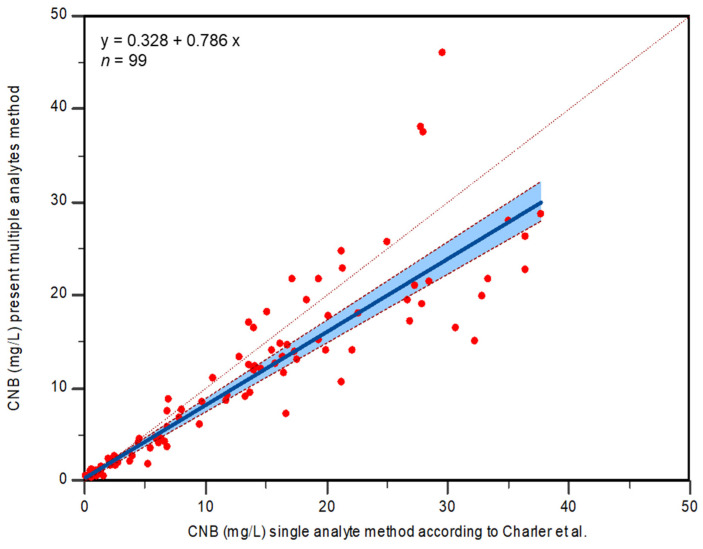
Passing–Bablok correlation plot of CNB plasma concentrations (mg/L) obtained with reference method [23] (*X*-axis) compared with those obtained with the method here presented (*Y*-axis). Thick line, regression line; thin line, identity line; dashed line, confidence interval for the regression line.

The performance of the method was evaluated by analyzing 99 patient samples, and the results are shown in the Passing–Bablok regression plot [slope with 95% confidence interval (CI) and intercept with 95% CI] (Figure 5). Regression analysis (regression equation y = 0.328472 + 0.785880x) showed an intercept of 0.3285 (95% CI, 0.01928–0.4516) and a slope of 0.7859 (95% CI, 0.7419–0.8434). No significant deviation from linearity was observed (*p* = 0.51). The correlation coefficient was 0.961 with a 95% CI (0.942–0.973) with *p* < 0.0001.

**Figure 6:**
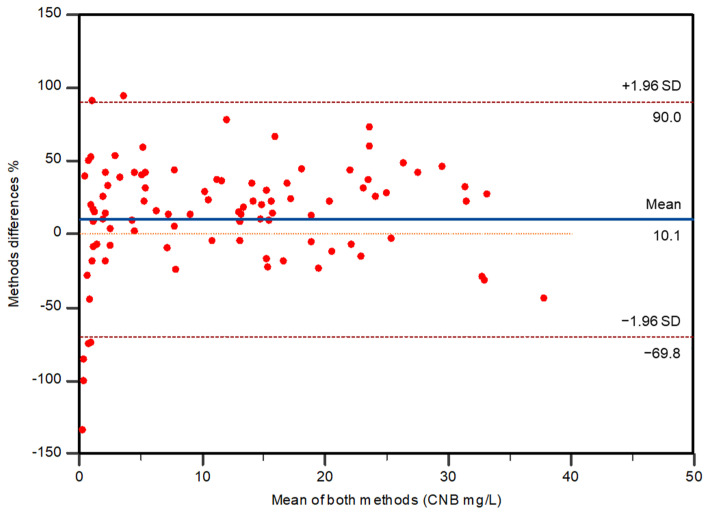
Bland–Altman plot includes the reference line for no difference (at 0 mg/L) (dotted line), the solid line indicating mean % difference and the 95% limits of agreement of the methods (dashed lines).

The Bland–Altman plot (bias with 95% CI, 95% limits of agreement) (Figure 6) showed a mean bias of 10.1% (95% CI, 1.9405–18.2033). 

CNB concentrations in patients’ samples, when determined with the reference method, ranged from 0.1 to 37.7 mg/L (mean 12.3 mg/L, median 10.6 mg/L), while the present method varied from 0.4 to 46.0 mg/L (mean 10.42 mg/L, median 8.7 mg/L).

## 3. Materials and Methods

### 3.1. Chemicals and Reagents

Cenobamate (racemic mixture) was purchased from Alsachim (Illkirch, France). A stock solution was prepared in water at the nominal concentration of 0.8 mg/mL and stored at −20 °C.

Ultrapure water was obtained from a Milli-Q water purification system (Millipore, Milan, Italy). LC-MS/MS grade acetonitrile and methanol were purchased from Merck (Milan, Italy).

As reference method, a commercial CE/IVD kit for the quantification of antiepileptic drugs in human serum/plasma (“Plasmatic AEDs in the LC-MS—Deuterated Internal Standards”) was purchased from Eureka Lab Division, a division of Sentinel Diagnostic (Milan, Italy).

### 3.2. Calibration Curves and Quality Control Samples

Calibration curves and QC samples were obtained by reconstituting the lyophilized material provided by the commercial kit with 1 mL of Milli-Q water, following the manufacturer instructions. CNB was added to the highest calibration standard, which was then serially diluted with blank plasma from healthy donors to obtain a seven-point calibration curve (0.0, 2.4, 5.0, 10.0, 20.0, 40.0, and 80.0 mg/L). To obtain QC samples, CNB was added to the fifth level of the blank calibration curve, which was then serially diluted with blank plasma from healthy donors to obtain 3 levels of QCs, defined as LQC, MQC, and HQC (3.0, 30.0, and 60.0 mg/L). Nominal concentration values of the calibration curve and QC samples for the other detected compounds are shown in Supplementary Material (Table S1).

### 3.3. Sample Preparation

#### 3.3.1. Manual Sample Preparation

Fresh whole blood was collected by venipuncture in lithium-heparin-containing tubes; after collection, tubes were centrifuged at 3500× *g* for 15 min and plasma was collected. Plasma samples were either processed or stored at −20 °C until further analysis.

Samples were extracted by following the analytical procedure indicated by the kit manufacturer. A total of 50 µL of sample was dispensed into a 1.5 mL tube and 25 µL of mobile phase A (Water, 0.1% HCOOH) was added; the sample underwent a protein precipitation step by adding 500 µL of acetonitrile (ACN) spiked with IS to the tube (IS are listed in Table S5). Samples were then vortex mixed and incubated at −20 °C for 15 min. The tubes were then centrifuged at 16,000× *g* for 15 min; 50 µL of supernatant was added to 450 µL of mobile phase A in an autosampler vial. A total of 1 µL of the obtained solution was injected into the instrument.

#### 3.3.2. Automated Sample Preparation

In order to automatize and standardize the sample preparation procedure, the samples were also extracted by employing a Tecan Freedom EVO 100 liquid-handling unit equipped with Freedom EVOware Version 2.8 SP2 software (Tecan Italia S.r.l., Cernusco sul Naviglio, Italy). 

A sample of 50 µL was dispensed into a 96-well plate and 25 µL of mobile phase A (Water, 0.1% HCOOH) was added; the sample underwent a protein precipitation step by adding 500 µL of ACN spiked with IS (IS are listed in Table S5). The plate was then mixed by vortexing, incubated at −20 °C for 15 min, and then centrifuged at 3500× *g* for 20 min; subsequently, 50 µL of supernatant was added to 450 µL of mobile phase A in a clean plate. A total of 1 µL of the obtained solution was injected into the instrument.

### 3.4. Instrument Setup and Parameters

LC-MS/MS analysis was carried out on a Waters Xevo TQ-XS triple quadrupole mass spectrometer coupled to a Waters Acquity UPLC I-Class System (Waters Corporation, Sesto San Giovanni, Italy). Chromatographic separation was performed on an Agilent InfinityLab Poroshell EC-C18 column (2.1 × 50 mm, 1.9 µm particle size) (Agilent, Santa Clara, CA, USA) with two mobile phases, A (Water, 0.1% HCOOH) and B (ACN, 0.1% HCOOH). Elution was performed in gradient elution mode: at a flow rate of 0.5 mL/min, the gradient was stepped from 2% B to 13% B in 0.5 min and held for 1.3 min; then, it was stepped to 21% B in 0.1 min and held for 1 min, stepped to 50% B in 0.5 min and held for 0.5 min, and stepped to 80% B in 0.1 min and held for 0.3 min. Column was washed at 95% B for 0.6 min and restored to the initial condition of 2% B for column equilibration. Total run time was 7 min; column oven was set at 35 °C.

MS analysis was performed using a triple quadrupole mass spectrometer equipped with an electrospray ionization (ESI) source operated both in positive and negative ion mode. Method parameters were the following: source temperature 150 °C, capillary voltage +1.5 kV, desolvation temperature 500 °C, cone gas flow rate 150 L/h, collision gas flow rate 0.15 mL/min, and desolvation gas flow rate 1000 L/h. Dwell time was automatically calculated. Waters MassLynx (V 4.2) and TargetLynx (V 4.2) software was used to acquire and process data (Waters Corporation, Sesto San Giovanni, Italy).

All compounds were detected in multiple reaction monitoring (MRM) mode as described in Supplementary Material (Table S5). The detecting conditions for CNB were obtained by directly infusing the standard solution at 100 ng/mL and were the following: precursor ion 268.02 *m*/*z*, cone voltage 20 V, quantifier ion 154.99 *m*/*z* (collision energy 14 V), qualifier ion 197.99 *m*/*z* (collision energy 10 V), ion mode ES+, retention time 3.32 min.

Similarly to our previous study, available deuterated standards such as felbamate-d4, topiramate-d12, and lamotrigine-13C-d3 were tested to be employed as IS. Lamotrigine-13C-d3 was found to provide better results, both in terms of chromatographic resolution and recovery, and showed good normalization capabilities [23]. 

### 3.5. Method Validation

Method validation for CNB was performed according to the most recent ICH Guideline M10 for Bioanalytical Method Validation and Study Sample Analysis [24], which requires the following assays: selectivity and specificity, calibration curve linearity, carry-over, dilution integrity, matrix effect, accuracy and precision, reinjection reproducibility, stability, and incurred sample reanalysis. A partial validation was performed on the modified kit in order to assess its robustness and reliability in the monitoring of concomitant ASMs.

#### 3.5.1. Selectivity and Specificity

Method selectivity and specificity were assessed by analyzing six different lots of blank matrices, including (i) reconstituted highest calibration standard (without CNB), (ii) two matrices of blank plasma from healthy donors, and (iii) three matrices of Li-Heparin plasma containing other ASMs. The absence of significant response accountable to interfering components at analyte retention time was assessed; response should not be greater than 20% of analyte response at LLOQ.

#### 3.5.2. Linearity

Calibration curves were built by plotting analyte/IS peak area ratio against corresponding analyte nominal concentrations and fitted by using a 1/x weighted linear regression model.

To assess the LLOQ, CNB-spiked plasma samples at concentrations of 2, 0.8, 0.5, and 0.4 mg/L were prepared and analyzed in triplicate. LLOQ was defined as the lowest concentration level at which accuracy and precision were within ±20%.

#### 3.5.3. Carry-Over

Carry-over was assessed by analyzing six blank plasma samples from healthy donors after the injection of the highest calibration standard. Response should not be greater than 20% of analyte response at LLOQ.

#### 3.5.4. Dilution Integrity

Dilution integrity was performed to assess the feasibility of the dilution procedure. A high-concentrated plasma sample (120 mg/L CNB) was serially diluted with blank plasma to obtain 1:2 and 1:16 dilutions falling within the calibration range. Diluted samples were analyzed in triplicate. The mean accuracy and precision of diluted samples should be within ±15%.

#### 3.5.5. Recovery and Matrix Effect

RE and ME for CNB were assessed at two concentration levels (LQC and HQC) by analyzing seven different lots of matrices in triplicate, including (i) lipemic Li-Heparin plasma containing other ASMs, (ii) normal Li-Heparin plasma containing other ASMs, (iii) hemolyzed Li-Heparin plasma containing other ASMs, (iv) three matrices of blank plasma from healthy donors, and (v) reconstituted blank calibration standard. RE and ME were calculated according to Matuszewski [29]; IS normalization was applied as described in De Nicolò et al. [30], thus, ISn-ME and Isn-RE values are shown.

#### 3.5.6. Accuracy and Precision

Intra-day and inter-day accuracy and precision were assessed at four concentration levels (LLOQ, LQC, MQC, and HQC) with a 5 × 5 scheme, consisting of five replicates for each concentration level in five analytical runs over five non-consecutive days.

Accuracy is expressed as relative error, or bias%, which is calculated as the percentage difference between measured and nominal concentrations, while precision is expressed as CV%. Mean accuracy and precision values within ±15% for LQC, MQC, and HQC, and within ±20% for LLOQ, were considered acceptable.

#### 3.5.7. Reinjection Reproducibility

Reinjection reproducibility was assessed to establish the viability of processed samples. One run from accuracy and precision experiments was reinjected after storage in the autosampler compartment (10 °C) for 24 h and accuracy and precision of reinjected QCs were assessed.

#### 3.5.8. Stability

Sample stability was measured at two concentration levels (LQC and HQC) in triplicate. Short-term stability was assessed at RT for 72 h and at 4 °C for 48 h, 72 h, and 1 week; autosampler stability was assessed by storing extracts in the autosampler compartment at 10 °C for 24 h. Medium-term stability at −40 °C for 1 month and freeze-and-thaw (FT) stability at −20 °C were assessed.

The mean concentration values obtained from each measurement were compared to the concentrations measured at T0 before storage. Stability data are expressed as percentage difference from T0.

#### 3.5.9. Incurred Sample Reanalysis

ISR is aimed at assessing the accuracy of the reported analyte concentration in study samples, since they may differ from calibration standards due to matrix issues. 

From 29 study samples, 15 samples were reanalyzed. The percentage difference between the initial concentration and the concentration measured during ISR was calculated as the percentage ratio between the difference of repeated and initial value and the mean value. 

For at least 2/3 of the repeats the percentage difference should be within ±20%.

#### 3.5.10. Automated Sample Extraction Validation

The method was also partially validated for CNB when an automated sample extraction procedure was employed, as described in Section 3.3.2., according to the most recent ICH Guideline for Bioanalytical Method Validation [24]. Carry-over, RE, and ME were assessed.

Carry-over was assessed by analyzing six blank plasma samples from healthy donors after the dispensation of the highest calibration standard. Response should not be greater than 20% of analyte response at LLOQ.

RE and ME were assessed at LQC and HQC by analyzing seven different lots of matrices in triplicate, including (i) lipemic Li-Heparin plasma containing other ASMs, (ii) normal Li-Heparin plasma containing other ASMs, (iii) hemolyzed Li-Heparin plasma containing other ASMs, (iv) three matrices of blank plasma from healthy donors, and (v) reconstituted blank calibration standard. RE and ME were calculated according to Matuszewski [29]; IS normalization was applied as described in De Nicolò et al. [30], thus, IS normalized ME (ISn-ME) and IS normalized RE (ISn-RE) values are shown.

### 3.6. Method Comparison

Finally, to assess the robustness and validity of the new method, the concentration of 99 samples obtained from the three hospitals participating in this study (Fondazione IRCCS Istituto Neurologico Carlo Besta, Milano; Azienda Ospedaliero-Universitaria “San Giovanni di Dio e Ruggi d’Aragona-Scuola Medica Salernitana”, Salerno; Fondazione Policlinico Universitario Campus Biomedico, Roma) were compared with those obtained with the previously published method. Patients are described in the following table (Table 5).

#### Statistical Analysis

Method cross-validation was performed considering the previously published LC-MS/MS assay as the reference method and the one here described as the test method. Methods were compared by Passing–Bablok regression and Bland–Altman plot, obtained with MedCalc Statistical Software version 22.014 (MedCalc Software, Mariakerke, Belgium) and MetComp Software version 1 (GdS SIBioC, Novara, Italy) [31].

## 4. Conclusions

As a result of CNB’s recent approval as an ASM, limited information is available regarding its plasmatic therapeutic ranges and its real-life interactions with other concomitant drugs. Also, since the titration process represents a crucial step for drug safety [16], the determination of plasma concentrations could provide the clinician with useful information for the identification of tailor-made therapeutic regimens during both the titration and maintenance periods. 

For this reason, we provide the development and validation of a UHPLC–MS/MS quantification method for the detection of CNB and other ASMs, starting from a commercially CE/IVD validated kit for the quantification of antiepileptic drugs in human plasma and serum. Our method was validated for CNB but also allowed the detection and quantification of other drugs, which are often administered in co-therapy with CNB. The simultaneous detection of 17 compounds may represent a saving in terms of both reagent costs and time, and provides a more efficient clinical management of the patient. 

Overall, our method showed a good performance, with a simple extraction procedure and a relatively short run time for the detection of a total of 17 compounds. The absence of interferences between CNB and the other compounds within the analytical determination is of crucial importance for the introduction of CNB into routine TDM of ASMs. 

Future studies will be needed in order to improve our understanding of CNB’s pharmacological features and interactions, and for the determination of a therapeutic reference range aimed at identifying personalized drug regimens and ensuring drug clinical efficacy and safety.

## Figures and Tables

**Table 1:** Comparison of existing analytical methods for the quantification of CNB in LC-MS/MS. EDTA = ethylenediamine tetraacetic acid; AcN = acetonitrile; MeOH = methanol; APCI = atmospheric pressure chemical ionization; ESI = electrospray ionization.

Methods	Calibration Range for CNB	Co- Detected Compounds	Matrix (Anticoagulant)	Extraction	Internal Standard	Chromatographic Separation			Mass Spectrometry Analysis	
						Column	Mobile Phase	Run Time	Instrument	Source
Drug Approval Package: XCOPRI [22]	0.02–10.00 mg/L	/	Human plasma (heparin)	Protein precipitation	Phenacetin	/	/	/	AB/MDS Sciex API 4000 (triple quadrupole)	/
Oh et al., 2019 [25]	0.01–5.00 mg/L	/	Rat plasma	Protein precipitation (AcN)	Carisbamate (30 mg/L)	C18 column	60:40 (*v*/*v*) ammonium formate (10 mM):AcN	3 min	AB/MDS Sciex API 4000 (triple quadrupole)	ESI
Vernillet et al., 2020 [26]	0.02–25.00 mg/L	/	Human plasma (sodium heparin)	Protein precipitation (AcN)	Phenacetin (0.01 mg/L)	C18 column	62:38 (*v*/*v*) ammonium acetate (2 mM):MeOH	/	Triple quadrupole mass spectrometer	Heated nebulizer source
	0.02–10.00 mg/L	/		Protein precipitation (AcN), evaporation and reconstitution with 40:60 (*v*/*v*) AcN:H_2_O	Phenacetin (0.01 mg/L)	C18 column	62:38 (*v*/*v*) ammonium acetate (2 mM):MeOH	/	Triple quadrupole mass spectrometer	APCI
	0.05–40.00 mg/L	/		Lipid extraction (methyl tertiary-butyl ether), evaporation and reconstitution with 3:2 (*v*/*v*) MeOH:H_2_O	d4-cenobamate (100 mg/L)	/	A: 1000; 1.00; 0.40 (*v*/*v*) H_2_O:HCOOH:ammonium hydroxide B: 1000; 1.00; 0.40 (*v*/*v*) MeOH:HCOOH:ammonium hydroxide	/	Triple quadrupole mass spectrometer	TurboIon Spray^®^
Charlier et al., 2022 [23]	0.05–20.00 mg/L	/	Human plasma (EDTA)	Protein precipitation (AcN)	Lamotrigine-C13-d3 (2 mg/L)	Pentafluoro-phenyl column	A: H_2_O:0.1% HCOOH B: AcN:0.1% HCOOH	3.5 min	Endura TSQ (triple quadrupole)	ESI
Our Method	0.80–80.00 mg/L	Other 16 ASMs	Human plasma (lithium heparin)	Protein precipitation (AcN) and dilution with mobile phase	Lamotrigine-C13-d3	C18 column	M1: H_2_O:0.1% HCOOH M2: AcN:0.1% HCOOH	7 min	Xevo TQ-XS (triple quadrupole)	ESI

**Table 2:** Summary of matrix effect (ME) and recovery (RE) data and internal standard normalized (ISn) values (*n* = 7) for CNB. LQC = low QC (3 mg/L); HQC = high QC (60 mg/L); CV = coefficient of variation.

		Matrix 1	Matrix 2	Matrix 3	Matrix 4	Matrix 5	Matrix 6	Matrix 7
**ME%**	LQC	107	106	105	112	111	113	107
	HQC	102	103	109	111	114	111	112
**RE%**	LQC	105	101	106	105	105	99	106
	HQC	107	98	101	102	98	106	108
**ISn-ME%**	LQC	99	92	99	101	105	102	92
	HQC	90	97	98	99	99	101	100
**ISn-RE%**	LQC	112	112	101	106	99	97	111
	HQC	111	98	104	111	107	113	114

**Table 3:** Inter-day and intra-day precision and accuracy of the quantification method in human plasma. QCs = quality control samples; LLOQ = lower limit of quantification (0.8 mg/L); LQC = low QC (3 mg/L); MQC = medium QC (30 mg/L); HQC = high QC (60 mg/L); CV = coefficient of variation.

		INTER-DAY					INTRA-DAY
QCs		Day 1	Day 2	Day 3	Day 4	Day 5	
LLOQ	Bias%	7.2	18.4	−11.9	−16.3	−6.7	−1.9
	CV%	4.4	2.6	10.2	9.2	3.4	14.6
LQC	Bias%	5.4	−1.7	−0.9	4.5	12.4	3.9
	CV%	3.4	3.2	5.2	7.4	6.1	5.5
MQC	Bias%	−0.6	−5.3	10.3	1.4	4.8	2.1
	CV%	6.1	5.3	5.8	9.3	7.8	5.8
HQC	Bias%	−0.2	−13.7	−0.5	−1.2	−1.5	−3.4
	CV%	3.6	5.5	6.5	3.2	7.1	6.0

**Table 4:** Short-term and medium-term stability in plasma samples and extracts for different storage times and conditions (*n* = 3). Stability is expressed as percentage difference from T0 (% of degradation). LQC = low QC (3 mg/L); HQC = high QC (60 mg/L).

Storage Conditions	QCs	% Difference from T0		
		48 h	72 h	1 week
4 °C	LQC	10.6	−14.6	−11.6
	HQC	−0.8	−11.3	−13.6
		**72 h**		
RT	LQC	−27.8		
	HQC	−19.6		
		**24 h**		
Autosampler (10 °C)	LQC	−7.9		
	HQC	−0.6		
		**1 month**	**Freeze-thaw**	
−20 °C	LQC	−19.5	−16.5	
	HQC	5.1	−9.6	
		**1 month**		
−40 °C	LQC	−11.9		
	HQC	−3.4		

**Table 5:** Summary of patients’ data used for method comparison (*n* = 47, samples *n* = 99).

Gender		
	Woman	16 (34%)
	Man	31 (66%)
Age (year), median (range)		26.21 (18–60)
Body weight (kg), median (range) (*n* = 37)		66.62 (54–107)
Cenobamate dose (mg) (*n* = 86)		
	Mean (SD)	115 (81)
	Median (range)	100 (12.5–250)
Cenobamate plasma concentration (mg/L)		
	Mean (SD)	10 (10)
	Median (range)	7 (0.4–46)
Patients with no data		7
Patients with unknown concomitant ASMs		19
Patients with 1 concomitant ASM		4
Patient with 2 or more concomitant ASMs		17

## Data Availability

Data are contained within the article and supplementary materials.

## Supplementary Materials

The following supporting information can be downloaded at: https://www.mdpi.com/article/10.3390/molecules29040884/s1, Table S1: Nominal concentrations of calibration curves and quality control (QC) samples; Table S2: Intra-day precision and accuracy of the quantification method in human plasma for the detection of the other detected ASMs; Table S3: Inter-day precision and accuracy of the quantification method in human plasma for the detection of the other detected ASMs; Table S4: Demographic and therapeutic features of patients receiving CNB as co-ASMs in polytherapy at Fondazione IRCCS Istituto Neurologico Carlo Besta; Table S5: MS method transitions, parameters, and retention times.
