# Supplementary material for: Quantitative Analysis of Cenobamate and Concomitant Anti-Seizure Medications in Human Plasma via Ultra-High Performance Liquid Chromatography–Tandem Mass Spectrometry
Source: Molecules. 2024 Feb 17;29(4):884. doi: 10.3390/molecules29040884 (PMC10892084; doi:10.3390/molecules29040884)
Supplement: Supplementary file 1 [file molecules-29-00884-s001.zip › molecules-2795843-supplementary.pdf]

Supplementary Material

Table S1. Nominal concentrations of calibration curves and quality control (QC) samples. Concentrations are expressed in mg/L, except for TGB (ng/mL). Compounds are listed in order of retention time apart from CNB. CAL0 = calibrator 0, CAL1 = calibrator 1, CAL2 = calibrator 2, CAL3 = calibrator 3, CAL4 = calibrator 4, CAL5 = calibrator 5, CAL6 = calibrator 6, LLOQ = lower limit of quantification, LQC = lower QC, MQC = medium QC, HQC = high QC, CNB = cenobamate, PGB = pregabalin, GBP = gabapentin, LEV = levetiracetam, ETS = ethosuximide, LTG = lamotrigine, PRM = primidone, LCS = lacosamide, ZNS = zonisamide, RUF = rufinamide, MHD = 10-monohydroxy carbazepine, BRV = brivaracetam, CBZ-EP = carbamazepine-epoxide, TPM = topiramate, TGB = tiagabine, PMP = perampanel, STP = stiripentol.

Compound	CAL0	LLOQ	CAL1	CAL2	CAL3	CAL4	CAL5	CAL6	LQC	MQC	HQC
**CNB**	0.00	0.80	2.40	5.00	10.00	20.00	40.00	80.00	3.00	30.00	60.00
**PGB**	0.00	0.01	0.04	0.08	0.16	0.33	0.65	1.30	0.04	0.04	0.08
**GBP**	0.00	0.19	0.56	1.17	2.34	4.68	9.35	18.70	0.61	6.10	12.20
**LEV**	0.00	0.5	1.50	3.13	6.26	12.53	25.05	50.10	1.53	15.30	30.60
**ETS**	0.00	1.10	3.29	6.84	13.69	27.38	54.75	109.50	3.41	34.05	68.10
**LTG**	0.00	0.20	0.59	1.23	2.45	4.90	9.80	19.60	0.61	6.10	12.20
**PRM**	0.00	0.25	0.76	1.58	3.16	6.33	12.65	25.30	0.82	8.20	16.40
**LCS**	0.00	0.19	0.58	1.21	2.43	4.85	9.70	19.40	0.61	6.10	12.20
**ZNS**	0.00	0.38	1.15	2.40	4.80	9.60	19.20	38.40	1.20	12.00	24.00
**RUF**	0.00	0.46	1.38	2.88	5.76	11.53	23.05	46.10	1.45	14.50	29.00
**MHD**	0.00	0.51	1.52	3.18	6.35	12.70	25.40	50.80	1.58	15.80	31.60
**BRV**	0.00	0.06	0.19	0.39	7.90	1.58	3.15	6.30	0.22	2.20	4.40
**CBZ-EP**	0.00	0.08	0.23	0.48	0.96	1.93	3.85	7.70	0.25	2.45	4.90
**TPM**	0.00	0.28	0.83	1.73	3.46	6.93	13.85	27.70	0.90	9.00	18.00
**TGB**	0.00	0.40	1.20	2.50	5.00	10.00	20.10	40.10	1.30	13.00	26.00
**PMP**	0.00	0.03	0.08	0.16	0.33	0.65	1.30	2.60	0.08	0.75	1.50
**STP**	0.00	0.28	0.85	1.76	3.53	7.05	14.10	28.20	0.87	8.65	17.30

Table S2. Intra-day precision and accuracy of the quantification method in human plasma for the detection of the other detected ASMs. LLOQ = lower limit of quantification, LQC = lower QC, MQC = medium QC, HQC = high QC, CNB = cenobamate, PGB = pregabalin, GBP = gabapentin, LEV = levetiracetam, ETS = ethosuximide, LTG = lamotrigine, PRM = primidone, LCS = lacosamide, ZNS = zonisamide, RUF = rufinamide, MHD = 10-monohydroxy carbazepine, BRV = brivaracetam, CBZ-EP = carbamazepine-epoxide, TPM = topiramate, TGB = tiagabine, PMP = perampanel, STP = stiripentol.

		PGB					GBP					LEV				
		**Day1**	**Day2**	**Day3**	**Day4**	**Day5**	**Day1**	**Day2**	**Day3**	**Day4**	**Day5**	**Day1**	**Day2**	**Day3**	**Day4**	**Day5**
**LLOQ**	**Bias%**	10.0	18.0	-10.0	2.0	10.0	0.7	5.7	-18.0	-16.8	-9.1	4.3	2.7	14.4	-16.6	-4.8
	**CV%**	6.4	9.3	11.1	8.2	9.1	3.7	3.7	8.6	4.2	3.4	1.3	7.4	6.4	6.6	3.9
**LQC**	**Bias%**	-11.0	-6.0	-2.5	-3.0	1.5	-3.4	-7.9	-13.7	8.3	12.3	11.2	2.4	4.3	11.8	14.9
	**CV%**	7.6	9.7	10.4	5.6	5.9	2.8	3.5	9.0	5.0	3.8	5.9	7.8	4.9	7.6	2.5
**MQC**	**Bias%**	2.1	6.8	11.7	-0.1	-1.3	-7.3	-4.5	1.3	-1.9	-5.5	1.1	10.5	10.6	2.7	1.1
	**CV%**	5.3	3.1	8.2	5.2	4.1	4.6	3.5	5.1	3.1	2.9	2.2	3.5	3.7	2.8	4.7
**HQC**	**Bias%**	0.6	4.4	8.5	1.5	0.2	-7.3	-7.8	-1.3	-6.2	-9.9	2.9	10.0	8.8	-1.9	-4.0
	**CV%**	3.1	2.0	2.4	1.3	3.4	3.2	2.6	3.9	2.1	2.8	4.5	3.7	6.2	4.4	2.6

		ETS					LTG					PRM				
		**Day1**	**Day2**	**Day3**	**Day4**	**Day5**	**Day1**	**Day2**	**Day3**	**Day4**	**Day5**	**Day1**	**Day2**	**Day3**	**Day4**	**Day5**
**LLOQ**	**Bias%**	1.4	10.6	-10.7	-11.2	-10.2	8.2	2.6	-18.3	-15.1	-3.1	6.8	-6.1	-13.0	-12.1	11.5
	**CV%**	2.7	12.3	8.4	6.4	10.3	8.2	11.3	4.7	7.6	2.6	11.6	12.9	11.3	4.3	3.1
**LQC**	**Bias%**	1.6	1.2	4.3	3.6	5.2	-1.3	-5.2	5.3	13.1	9.7	-2.6	-5.9	5.0	8.1	11.1
	**CV%**	4.6	3.3	9.0	6.5	6.7	4.5	2.2	4.5	6.1	5.6	10.3	5.0	7.6	6.4	11.5
**MQC**	**Bias%**	-5.6	1.7	-1.0	-8.2	0.2	-5.2	2.3	2.8	3.0	-4.6	-8.0	-9.3	-2.9	-9.1	-3.3
	**CV%**	7.2	4.2	4.7	6.2	3.0	2.4	5.4	9.4	4.5	3.6	4.8	6.9	9.0	4.6	4.5
**HQC**	**Bias%**	-3.9	-2.0	1.4	-9.7	-9.3	-5.9	-3.7	0.3	-1.2	-5.6	-7.4	-7.5	-8.2	-9.3	-2.3
	**CV%**	2.6	3.3	8.0	3.2	3.5	2.4	2.9	5.0	4.4	3.0	2.9	3.7	4.1	8.0	5.5

		LCS					ZNS					RUF				
		**Day1**	**Day2**	**Day3**	**Day4**	**Day5**	**Day1**	**Day2**	**Day3**	**Day4**	**Day5**	**Day1**	**Day2**	**Day3**	**Day4**	**Day5**
**LLOQ**	**Bias%**	13.7	14.2	-6.3	-9.6	-16.8	-5.8	5.2	-6.5	-18.3	-18.7	10.8	10.5	-4.1	-14.8	4.8
	**CV%**	8.1	6.2	8.0	4.6	6.8	4.2	4.0	6.3	7.2	2.2	8.8	3.2	4.4	5.5	2.3
**LQC**	**Bias%**	-1.7	-6.2	1.2	9.4	7.8	-4.3	-6.0	3.9	4.9	10.3	12.6	4.8	7.8	13.3	11.8
	**CV%**	6.4	2.9	6.9	8.0	3.1	0.8	3.7	5.3	5.3	6.6	7.3	4.0	6.7	7.5	3.0
**MQC**	**Bias%**	-7.0	-1.3	-4.3	-2.9	1.9	-4.0	-3.7	-0.8	-2.5	2.7	5.4	4.6	2.1	-0.3	0.6
	**CV%**	4.5	1.6	7.0	2.7	5.3	4.6	5.0	6.7	4.9	3.2	4.8	1.9	5.9	5.0	6.9
**HQC**	**Bias%**	-3.1	-1.5	-6.3	-4.3	-2.5	-3.0	-2.2	-2.4	-5.2	-1.9	9.7	3.4	0.1	-5.5	-3.7
	**CV%**	1.5	2.7	5.6	3.2	5.0	5.7	3.2	3.0	6.0	3.7	4.0	3.4	7.3	5.3	3.7

		MHD					BRV					CBZ-EP				
		**Day1**	**Day2**	**Day3**	**Day4**	**Day5**	**Day1**	**Day2**	**Day3**	**Day4**	**Day5**	**Day1**	**Day2**	**Day3**	**Day4**	**Day5**
**LLOQ**	**Bias%**	-2.9	-2.5	-4.4	-1.3	-9.6	15.4	18.1	-1.0	-10.2	-19.3	-10.0	-7.0	-0.5	-12.5	-9.5
	**CV%**	1.4	10.7	6.1	3.7	4.9	14.6	9.7	9.2	11.1	7.9	4.6	11.7	6.5	5.9	8.1
**LQC**	**Bias%**	-4.4	-14.1	1.8	11.9	5.5	1.0	-13.3	-4.8	-0.2	-3.3	-9.8	-10.2	4.3	2.9	6.7
	**CV%**	5.9	6.5	4.0	10.8	2.8	4.3	6.4	10.6	3.1	5.5	9.0	7.7	7.7	7.8	5.5
**MQC**	**Bias%**	-2.0	-0.9	0.6	1.3	-0.1	-13.0	-9.1	-8.7	-12.1	-6.0	-11.5	2.1	-8.0	-0.2	0.7
	**CV%**	7.3	2.1	4.7	4.4	3.7	2.1	3.7	3.8	2.5	7.3	8.5	5.4	4.7	6.7	8.6
**HQC**	**Bias%**	-4.7	-2.8	2.3	-1.8	-4.2	-12.1	-11.2	-7.7	-12.5	-7.1	-11.4	-4.2	-10.1	-3.0	-10.5
	**CV%**	3.4	3.2	4.4	4.0	3.1	1.9	4.1	9.0	7.0	3.6	8.8	2.3	13.9	5.2	6.4
		**TPM**					**TGB**					**PMP**				
		**Day1**	**Day2**	**Day3**	**Day4**	**Day5**	**Day1**	**Day2**	**Day3**	**Day4**	**Day5**	**Day1**	**Day2**	**Day3**	**Day4**	**Day5**
**LLOQ**	**Bias%**	-5.2	-2.0	-10.8	-10.4	-6.4	8.0	-6.5	-17.4	-6.0	-16.7	-17.7	-6.2	8.2	-13.3	-1.6
	**CV%**	9.5	3.4	4.9	11.3	5.0	3.5	7.2	4.6	2.0	3.2	6.0	7.5	6.4	3.8	2.2
**LQC**	**Bias%**	-2.6	-12.2	-1.1	7.2	11.2	-7.0	-7.7	0.5	1.6	5.5	-3.5	1.5	-8.9	12.8	2.6
	**CV%**	8.1	6.9	8.3	10.5	13.7	3.8	4.9	5.1	5.3	5.5	2.2	7.2	8.9	2.3	2.3
**MQC**	**Bias%**	-3.0	-2.6	-0.2	0.0	-2.8	-6.1	-2.3	-2.9	-12.3	-3.3	-1.7	7.5	8.7	1.6	6.2
	**CV%**	4.7	9.3	6.7	7.2	9.1	4.4	4.3	4.7	4.8	3.3	3.3	4.0	3.5	5.6	2.3
**HQC**	**Bias%**	-3.2	-9.6	-9.0	-2.3	-11.6	-8.0	-4.7	-7.9	-13.3	-7.6	-2.2	3.7	6.9	-1.6	2.3
	**CV%**	7.2	8.6	8.8	6.9	9.0	2.4	2.4	4.8	3.7	4.9	1.3	3.1	5.0	4.7	5.4
		**STP**														
		**Day1**	**Day2**	**Day3**	**Day4**	**Day5**										
**LLOQ**	**Bias%**	2.4	7.9	-7.5	-7.4	9.8										
	**CV%**	2.1	5.9	17.2	12.6	12.7										
**LQC**	**Bias%**	-0.3	-5.9	1.8	6.8	13.1										
	**CV%**	3.0	6.8	5.4	6.4	8.1										
**MQC**	**Bias%**	-4.8	-0.5	-4.0	0.6	-3.1										
	**CV%**	3.6	1.9	8.0	3.7	5.2										
**HQC**	**Bias%**	-3.9	-1.7	-2.3	-0.6	-4.1										
	**CV%**	2.7	2.6	6.9	2.6	4.2										

Table S3. Inter-day precision and accuracy of the quantification method in human plasma for the detection of the other detected ASMs. LLOQ = lower limit of quantification, LQC = lower QC, MQC = medium QC, HQC = high QC, CNB = cenobamate, PGB = pregabalin, GBP = gabapentin, LEV = levetiracetam, ETS = ethosuximide, LTG = lamotrigine, PRM = primidone, LCS = lacosamide, ZNS = zonisamide, RUF = rufinamide, MHD = 10-monohydroxy carbazepine, BRV = brivaracetam, CBZ-EP = carbamazepine-epoxide, TPM = topiramate, TGB = tiagabine, PMP = perampanel, STP = stiripentol.

		**PGB**	**GBP**	**LEV**	**ETS**	**LTG**	**PRM**	**LCS**	**ZNS**	**RUF**	**MHD**	**BRV**
**LLOQ**	**Bias%**	6.0	-7.5	-4.0	-5.1	1.1	-2.6	-1.0	-8.8	1.4	-4.1	0.6
	**CV%**	10.0	11.4	10.1	12.0	8.1	11.5	14.3	10.9	10.7	3.4	16.0
**LQC**	**Bias%**	-4.2	-0.9	3.2	4.3	3.1	3.2	2.1	1.8	10.0	0.1	-4.1
	**CV%**	4.8	11.0	1.7	7.3	7.0	7.0	6.4	6.7	3.3	9.9	5.9
**MQC**	**Bias%**	3.8	-3.6	-2.6	-0.4	-2.4	-6.5	-2.7	-2.7	2.5	-0.2	-9.8
	**CV%**	5.2	3.5	4.3	4.2	2.3	3.4	3.4	2.8	2.4	1.3	3.1
**HQC**	**Bias%**	3.0	-6.5	-4.7	-3.2	-2.5	-7.0	-3.5	-2.9	0.8	-2.3	-10.1
	**CV%**	3.4	3.4	5.0	2.8	1.5	2.9	1.9	1.4	6.0	2.8	2.8
		**CBZ-EP**	**TPM**	**TGB**	**PMP**	**STP**						
**LLOQ**	**Bias%**	-7.9	-7.0	-10.9	-6.1	1.1						
	**CV%**	5.0	4.0	3.6	10.8	8.1						
**LQC**	**Bias%**	-1.2	0.5	-1.4	0.9	3.1						
	**CV%**	8.2	9.1	5.8	8.0	7.0						
**MQC**	**Bias%**	-3.4	-1.7	-5.4	4.5	-2.4						
	**CV%**	6.2	1.5	4.4	4.2	2.3						
**HQC**	**Bias%**	-7.8	-7.1	-8.3	1.8	-2.5						
	**CV%**	4.3	4.4	3.4	3.7	1.5						

Table S4. Demographic and therapeutic features of patients receiving CNB as co-ASMs in polytherapy at Fondazione IRCCS Istituto Neurologico Carlo Besta. CNB = cenobamate, LEV = levetiracetam, LTG = lamotrigine, PRM = primidone, LCS = lacosamide, ZNS = zonisamide, BRV= brivaracetam, TPM = topiramate, PMP = perampanel, VPA = valproic acid, CBZ = carbamazepine, PB = phenobarbital, PHT = phenytoin.

Patient	Age (years)	Gender	CNB dose (mg/day)	CNB dose (mg/kg/day)	Co-therapy
Patient 1	25	M	50	0.83	VPA, CBZ
			200	3.28	VPA, CBZ
			250	4.17	VPA
			250	4.31	VPA
			250	4.24	VPA
Patient 2	56	F	200	3.64	PB, TPM
			350	6.36	TPM
			400	7.14	TPM
			400	7.14	TPM
Patient 3	20	M	25	0.43	BRV, LCS, PMP, clobazam
			250	4.31	BRV, LCS
			275	4.82	BRV, LCS
			300	5.45	BRV, LCS
Patient 4	59	F	250	3.62	PB, BRV, LCS
			300	4.35	PB, BRV, LCS
Patient 5	39	F	200	4.00	CBZ, BRV, clobazam
			200	3.85	CBZ, BRV
Patient 6	57	F	200	3.03	LEV, PGB, LCS
Patient 7	33	M	25	0.29	LTG, LCS
			150	1.76	LTG, LCS
Patient 8	37	M	50	0.71	VPA, PB, LCS
			150	2.08	VPA, PB, LCS
Patient 9	39	F	12.5	0.11	PB, LEV, PMP
			150	1.19	PB, LEV, PMP
Patient 10	27	M	100	1.56	PHT, PB, PRM
Patient 11	75	F	25	0.56	CBZ, ZNS
Patient 12	46	M	200	2.86	BRV, TPM
Patient 13	62	M	150	1.69	LEV, LCS
Patient 14	30	M	200	3.08	BRV, LTG

Table S5. MS method transitions (in bold **quantifier ion**, in italic *qualifier ion*), parameters and retention times. Compounds are listed in order of retention time apart from CNB. CNB = cenobamate, PGB = pregabalin, GBP = gabapentin, LEV = levetiracetam, ETS = ethosuximide, LTG = lamotrigine, PRM = primidone, LCS = lacosamide, ZNS = zonisamide, RUF = rufinamide, MHD = 10-monohydroxy carbazepine, BRV = brivaracetam, CBZ-EP = carbamazepine-epoxide, TPM = topiramate, TGB = tiagabine, PMP = perampanel, STP = stiripentol.

Compound	Precursor ion (m/z)	Cone (V)	Product ion (m/z)	Collision (V)	Ion mode	Retention time (min)	Internal standard
CNB	268.02	20	**154.99**	14	+	3.32	Lamotrigine-13C3,d3
			197.99	10			
PGB	160.00	34	**142.00**	10	+	1.00	Pregabalin-d4
			83.00	14			
GBP	172.00	38	**154.00**	11	+	1.01	Pregabalin-d4
			137.00	16			
LEV	171.00	22	**126.10**	14	+	1.08	Levetiracetam-d6
			154.00	7			
ETS	139.90	45	**140.00**	5	-	1.45	Ethosuximide-d3
			41.90	20			
LTG	256.10	55	**211.10**	24	+	1.82	Lamotrigine-13C3,d3
			158.90	30			
PRM	219.00	25	**162.20**	12	+	1.97	Primidone-d5
			90.90	25			
LCS	251.10	20	**91.20**	25	+	2.13	Lacosamide-d3
			108.00	8			
ZNS	210.90	25	**118.90**	15	-	2.16	Zonisamide-d4
			147.00	10			
RUF	239.00	32	**126.90**	25	+	2.39	Lacosamide-d3
			100.90	8			
MHD	255.10	20	**237.20**	7	+	2.63	10-monohydroxy carbazepine-d10
			194.00	20			
BRV	213.10	25	**168.20**	17	+	2.82	Levetiracetam-d6
			55.00	35			
CBZ-EP	253.00	27	**210.00**	15	+	3.13	Oxcarbazepine-d4
			180.00	15			
TPM	337.97	48	**337.97**	3	-	3.33	Topiramate-d12
			77.85	32			
TGB	376.00	45	**247.00**	45	+	3.56	Tiagabine-d5
			110.90	45			
PMP	350.00	72	**219.10**	36	+	3.98	Tiagabine-d5
			247.00	28			
STP	217.20	35	**187.00**	15	+	4.22	Stiripentol-d9
			145.00	15			
Pregabalin-d4	164.10	34	**146.10**	10	+	0.99	
Levetiracetam-d6	177.00	22	**132.10**	14	+	1.08	
Ethosuximide-d3	142.90	45	**143.00**	5	-	1.44	
Lamotrigine-13C3,d3	262.10	55	**217.10**	24	+	1.82	
Primidone-d5	224.00	25	**167.00**	12	+	1.94	
Lacosamide-d3	254.20	20	**108.00**	10	+	2.10	
Zonisamide-d4	214.90	25	**122.90**	15	-	2.12	

10-monohydroxy carbazepine-d10	258.10	20	**240.20**	7	+	2.62
Oxcarbazepine-d4	257.00	30	**240.00**	12	+	3.33
Topiramate-d12	350.03	54	**350.03**	3	-	3.29
Tiagabine-d5	382.00	45	**253.00**	20	+	3.56
Stiripentol-d9	226.00	35	**196.00**	15	+	4.21
